# Atlanta Medico-Chirurgical Society

**Published:** 1878-04

**Authors:** 


### ATLANTA MEDICO-CHIRURGICAL ASSOCIATION.

FRIDAY EVENING, March 1.

President G. G. Crawford in the chair.

Dr. E. J. Roach reported two cases of diptheria in infants—twins—both affected with the characteristic symptoms of diphtheria, and one of them had symptoms of pneumonia in left lung. Both recovered under the chlorine mixture. This mixture is prepared as follows:

℞.—Potassæ chloratis, . . . . . ʒi.

Acidi muriatis,

Aquæ, . . . . . . . . aa℥i.

M.

From two to eight drops of this to a tablespoonful of water may be given every two hours. For children, proportionately weaker and smaller doses should be used, and may be sweetened with syrup.

Dr. J. J. Knott said that, in 1865, he had published an article on diphtheria relative to the efficiency of creasote as a remedy locally applied. He had used a solution of one drachm of creasote to one ounce of mucilage. Often, one single application to the inflamed parts will suffice to effect a cure. He had seen violent cases recover in forty-eight hours. If there are complications, they must, of course, be met by appropriate agents. But, so far as his observation had gone, he was led to regard diphtheria a local affection merely, confined to the throat and developed by atmospheric influences. If fever and constitutional symptoms existed in some cases, they were the result of the local irritation.

Dr. R. H. Lee said he could not accord with the views of Dr. Knott. Diphtheria was a disease exceedingly fatal, especially in its epidemic form. Trosseau proves it to be contagious. Its constitutional character was manifested by the malaise and other constitutional spymptoms preceding, in some instances, the local affection. Death may result from the blood poison in diphtheria, while in membranous croup, which is local, we have death from asphyxia. Tracheotomy may be more successful in croup than in diphtheria.

Dr. A. R. Alley said: Diphtheria is one of the oldest epidemic diseases of the human race. Even Homer and Hippocrates advanced views, which Bretennau first sought to prove, that the disease was known even in those times as a disease greatly to be feared. Such was the disease which the ancients were acquainted with, and I differ with Dr. Knott as to diphtheria being a local disease. From my experience with this fearful malady, and from what I have seen of it in its epidemic form, it is a blood disease, highly contagious and capable of being transmitted from person to person, and the contagion of this disease might be carried through the air, thereby producing a toxical condition which we denominate infection; or it may even be communicated by solid materials, such as furniture of the sick room, utensils, clothing or bed-linen. Children or susceptible persons coming in contact with or using these materials will certainly be affected. Its pathology was considered to be fibrinous exudation, but it may be clearly set forth that it is albuminoid exudation. As to treatment, I have used and seen used nearly all the best known remedies to relieve the throat symptoms, but they have proved insufficient to arrest the fearful ravages of this malignant disease. I have seen used, with good effect, in a few well-marked sporadic cases, the following:

℞.—Chromic acid, 4 to 10 grains to 1 ounce of water.

Apply three or four times a day to the throat.

In a majority of cases, I have seen very little benefit resulting from topical application to the throat, unless associated with constitutional remedies, such as iron, quinine, chlorate potassium and stimulants. Its prognosis must be regarded as very unfavorable, as the mortality is fearfully great.

Dr. Pinson remarked that he had seen but little of the disease, but had experienced it in his own person when a child. It developed suddenly after exposure, the throat symptoms being severe and well marked. He inclined to the local theory of the disease.

Dr. R. C. Word being called upon for his views, remarked that during the greater part of his professional life he had regarded the throat affection in diphtheria as but the local manifestation of a constitutional disease. And until the last few years a majority of the profession seemed to hold this view.

The bacterian theory, a short while ago, had many advocates, it being very plausibly urged that these vegetable parasites, by their local irritation, produced the inflammation and all the throat symptoms, and that by their absorption and entrance into the blood, or the absorption of some poison or septic matter occasioned by them, the constitutional symptoms were produced.

The success which has been attributed to carbolic acid, chlorate of potash and similar agents, known to be antiparasitic, seemed to strengthen and sustain this view of the subject. But when certain investigators announced that these fungi had been found in localities where the disease had not occurred, and had even been seen upon the gums of healthy persons, this bacterian theory lost ground, and the controversy as to the local or constitutional character of the disease is reopened.

A strong point in favor of its being primarily a constitutional disease, is the not unfrequent malaise, nausea, rigors, headache, and other constitutional symptoms that are felt before the appearance of the local anginose symptoms. And it is also asserted that in exceptional instances in this affliction, as with scarlet fever, when an epidemic is prevailing, cases occur having the characteristic fever, sequellæ, etc., and yet without the throat affection; and it is true of scarlet fever, at least, that during its prevalence exceptionable cases occur in which the throat symptoms and the eruption are exceedingly light or entirely absent, thus showing the constitutional character of a disease, which, but for these exceptions, might very reasonably be regarded as a local affection merely.

Again, observations upon the pathology of septicæmia indicate most decidedly that septic innoculation is not successful upon healthy tissue, and that septic bacteria, and septic organisms generally, flourish only upon unhealthy or decaying tissues, thus showing that bacteria may be the result rather than the cause of the ulcerated and offensive condition of the throat in diphtheritic patients.

Dr. Word stated that in the treatment of diphtheria he had had very fair success with the saturated solution of the chlorate of potash frequently administered and used also as a gargle. He also gave quinine, and believed that these remedies were useful both as topical and constitutional agents.

Dr. Olmsted: I cannot accept the conclusions of Dr. Knott concerning the pathology of diphtheria, as they seem to be at variance with the facts revealed by clinical experience.

If diphtheria be a local and not a constitutional disease, and if, as Dr. Knott claims, the formation of the peculiar diphtheritic membrane is the first step in the morbid process, and the cause of the development of the morbid constitutional symptoms always observed, how is it that we have such profound constitutional disturbance, the chill, the fever (and often great prostration of the vital powers), which usually usher in and precede the manifestation of the characteristic throat lesion? Which in fact, during the prevalence of epidemic diphtheria, are present in some well-marked cases, without the usual throat lesion being observed? The Doctor's assumption that these prodromic symptoms, which appear before the formation of the false membranes (so essential to his theory) are due to some irritation of the throat, caused, perhaps, by the initial process in the formation of the membrane, is a theoretical conception, which has little to support it, either from our clinical observations, or any facts which the closest observers of the anatomical appearances of the throat have been able to discover. Moreover, if, as Dr. Knott claims, the constitutional symptoms are produced by the passage of the air over the diseased membranes, conveying the morbid material to the lungs, from which it is absorbed into the circulation, we might reasonably expect to find always, or at least usually, anatomical appearances in the lungs, characteristics of diphtheria, and the presence of false membranes throughout the bronchial tract. Such is not the case, for while occasionally the false membranes may extend into the small bronchial tubes, as a rule they do not extend beyond the trachea. In conclusion, Dr. Olmsted believes that diphtheria is a constitutional disease, dependent upon the production of its peculiar, specific poison (the nature of which remains to be discovered) into the blood; that the chill, fever, etc., etc., indicate such infection, and the characteristic membranes which appear in the throat, or in other parts of the body, are simply the local expressions of a constitutional disease, and not a "cause." He also thinks that the decomposition, and other chemical changes, which take place in this membrane, may add a new source of constitutional infection, just as the absorption of any other septic material may do, but this is only a secondary effect.

Dr. T. S. Powell: I doubt that the cases reported by Dr.

Roach were genuine diphtheria. The cause which developed the pneumonic symptoms mentioned in the one case might have produced the apparent diphtheritic affection in both. He was, however, not prepared to say much experimentally in regard to diphtheria, having had the good fortune in a long practice to see and treat but few cases of this fearful disease. He believed it to be very imperfectly understood by the profession generally; medical men differ greatly in their opinions as to the cause, pathology and treatment of this affection. He admitted that Dr. Knott's opinion, as to its local character, was plausible and was vindicated by a respectable and enlightened portion of the profession. But he had always entertained the view that genuine diphtheria was constitutional, the local manifestations in the throat being positive evidences of disease in the blood. He had noticed that scrofulous and tuberculous constitutions were peculiarly liable to this disease, especially in miasmatic localities. In certain of its features and perhaps its nature it was not unlike erysipelas, and should be treated upon the same principle. A definite plan of treatment could not be given for every case. Individual peculiarities must be met by appropriate remedies, but constitutional treatment looking to the elimination of the materies morbi, he thought important, and in many cases indispensable. He had found quinine, iron, and chlorate of potash the most successful agents to meet these indications.—*Southern Medical Record.*

